# Supplementary material for: Analysis of Neuronal Excitability Profiles for Motor-Eloquent Brain Tumor Entities Using nTMS in 800 Patients
Source: Cancers (Basel). 2025 Mar 10;17(6):935. doi: 10.3390/cancers17060935 (PMC11940777; doi:10.3390/cancers17060935)
Supplement: Supplementary file 1 [file cancers-17-00935-s001.zip › TableS1.pdf]

Variable		Glioma (N = 456)	Metastasis (N = 185)	Benign (N = 141)	P	Average SMD
**Patient Charateristics**						
Female		198 (43%)	94 (51%)	76(54%)	0.047	0.14
Age (y)		51 (15)	61 (11)	46 (17)	<0.001	0.68
Antiepileptic Medication		258 (57%)	74 (40%)	53 (38%)	<0.001	0.26
Motor Deficit (BMRC ≤4)		133 (29%)	101 (55%)	22 (16%)	<0.001	0.58
**Tumor Location & Morphology**						
Motor Location	M1-TMS-Infiltration	137 (30%)	94 (51%)	42 (30%)	<0.001	0.29
	TTD (mm)	6.4 (6.2)	7.1 (7.8)	6.8 (7.0)	0.470	0.07
Dominant Hemisphere		190 (42%)	86 (47%)	73 (52%)	0.091	0.14
Tumor Volume (ml)		30 (29)	11 (11)	14 (19)	<0.001	0.59
Edema Volume (ml)		42 (46)	61 (43)	11 (24)	<0.001	0.90
Multifocal (≥2 Foci)		66 (15%)	90 (51%)	20 (14%)	<0.001	0.53
Tumor Recurrence		145 (32%)	22 (12%)	13 (9%)	<0.001	0.39
**NTMS Parameter**						
RMT	Sick (V/m)	96 (27)	102 (30)	98 (23)	0.066	0.14
	Healthy (V/m)	96 (22)	95 (21)	100 (21)	0.135	0.15
	Ratio (%)	102 (27)	109 (32)	100 (18)	0.015	0.21
	Ratio (Pathologic)	253 (61%)	106 (66%)	52 (46%)	0.004	0.27
Area	Sick (mm^2)	322 (234)	287 (203)	277 (192)	0.086	0.14
	Healthy (mm^2)	316 (251)	288 (212)	306 (224)	0.593	0.08
Amplitude	Sick (μV)	619 (608)	546 (604)	612 (541)	0.425	0.08
	Healthy (μV)	788 (796)	698 (772)	842 (636)	0.392	0.13
Latency	Sick (ms)	23.6 (2.4)	23.5 (2.2)	22.8 (1.8)	0.005	0.25
	Healthy (ms)	23.5 (1.8)	24.1 (2.3)	22.8 (1.7)	<0.001	0.45
