# Supplementary material for: Analysis of Neuronal Excitability Profiles for Motor-Eloquent Brain Tumor Entities Using nTMS in 800 Patients
Source: Cancers (Basel). 2025 Mar 10;17(6):935. doi: 10.3390/cancers17060935 (PMC11940777; doi:10.3390/cancers17060935)
Supplement: Supplementary file 1 [file cancers-17-00935-s001.zip › TableS2.pdf]

Variable		WHO Grade				IDH Status			1p19q Status			Glioma Entity			
		WHO 2 (N = 54)	WHO 3 (N = 106)	WHO 4 (N = 251)	SMD	Mutation (N = 190)	Wildtype (N = 221)	SMD	Codel. (N = 68)	No Codel. (N = 343)	SMD	Oligo (N = 68)	Astro (N = 122)	GBM (N = 221)	SMD
**Patient Charateristics**															
Female		29 (54%)	43 (41%)	110 (44%)	0.18	86 (45%)	96 (43%)	0.04	30 (44%)	152 (44%)	0.04	30 (44%)	56 (46%)	96 (43%)	0.03
Age (y)		41 (13)	43 (11)	57 (14)	0.80	42 (12)	59 (13)	1.40	47 (13)	52 (16)	0.34	47 (13)	39 (10)	59 (13)	1.14
Antiepileptic Intake		38 (70%)	73 (71%)	120 (48%)	0.32	129 (69%)	102 (46%)	0.48	41 (61%)	190 (56%)	0.11	41 (61%)	88 (73%)	102 (46%)	0.38
Motor Deficit (BMRC ≤4)		10 (19%)	13 (12%)	92 (37%)	0.39	26 (14%)	89 (40%)	0.63	11 (16%)	104 (30%)	0.34	11 (16%)	15 (12%)	89 (40%)	0.45
**Tumor Location & Morphology**															
Motor Location	M1-TMS-Infiltration	16 (30%)	33 (31%)	72 (29%)	0.04	55 (29%)	66 (30%)	0.02	21 (31%)	100 (29%)	0.04	21 (31%)	34 (28%)	66 (30%)	0.04
	TTD (mm)	6.2 (4.9)	5.8 (6.4)	6.8 (6.4)	0.09	5.9 (5.9)	7.0 (6.5)	0.17	6.2 (5.6)	6.6 (6.4)	0.06	6.2 (5.6)	5.8 (6.1)	7.0 (6.5)	0.13
Dominant Hemisphere		25 (46%)	42 (40%)	102 (41%)	0.09	80 (42%)	89 (40%)	0.04	24 (35%)	145 (42%)	0.14	24 (35%)	56 (46%)	89 (40%)	0.15
Tumor Volume (ml)		26 (27)	41 (38)	28 (24)	0.32	35 (34)	27 (24)	0.25	34 (33)	30 (28)	0.11	34 (33)	35 (35)	27 (24)	0.18
Edema Volume (ml)		9 (26)	18 (34)	59 (48)	0.86	20 (35)	61 (48)	0.96	21 (39)	46 (48)	0.58	21 (39)	20 (34)	61 (48)	0.64
Multifocal (≥2 Foci)		3 (6%)	11 (10%)	42 (17%)	0.24	16 (8%)	40 (18%)	0.29	5 (7%)	51 (15%)	0.24	5 (7%)	11 (9%)	40 (18%)	0.22
Tumor Recurrence		12 (22%)	43 (41%)	77 (31%)	0.27	71 (37%)	61 (28%)	0.21	23 (34%)	109 (32%)	0.04	23 (34%)	48 (39%)	61 (28%)	0.17
**NTMS Parameter**															
RMT	Sick (V/m)	99 (28)	97 (23)	95 (27)	0.11	97 (25)	95 (27)	0.07	92 (22)	96 (27)	0.06	97 (22)	97 (27)	95 (27)	0.05
	Healthy (V/m)	96 (18)	98 (22)	95 (24)	0.10	97 (21)	95 (23)	0.09	97 (21)	95 (23)	0.09	97 (21)	97 (22)	95 (23)	0.08
	Ratio (%)	104 (30)	101 (22)	102 (27)	0.08	101 (24)	102 (28)	0.04	101 (22)	102 (27)	0.04	102 (22)	101 (25)	102 (28)	0.03
	Ratio (Pathologic)	23 (45%)	54 (56%)	149 (64%)	0.26	92 (52%)	134 (65%)	0.27	32 (50%)	194 (61%)	0.23	32 (50%)	60 (54%)	134 (65%)	0.21
Area	Sick (mm^2)	392 (270)	290 (178)	313 (236)	0.29	317 (208)	319 (247)	0.01	328 (238)	315 (227)	0.06	328 (238)	310 (190)	319 (247)	0.06
	Healthy (mm^2)	273 (200)	339 (241)	312 (267)	0.19	313 (223)	315 (279)	0.01	354 (263)	305 (248)	0.19	354 (263)	290 (195)	315 (279)	0.18
Amplitude	Sick (μV)	751 (708)	721 (608)	540 (530)	0.23	712 (612)	529 (544)	0.32	697 (748)	602 (545)	0.15	697 (748)	720 (528)	529 (544)	0.22
	Healthy (μV)	635 (619)	906 (776)	807 (878)	0.24	811 (739)	806 (895)	0.005	657 (539)	841 (865)	0.26	657 (539)	894 (818)	807 (895)	0.22
Latency	Sick (ms)	22.9 (2.1)	23.6 (2.1)	23.6 (2.4)	0.23	23.3 (2.1)	23.7 (2.5)	0.17	23.3 (2.0)	23.6 (2.4)	0.09	23.3 (2.0)	23.3 (2.2)	23.7 (2.5)	0.12
	Healthy (ms)	22.9 (1.7)	23.4 (1.8)	23.7 (1.7)	0.33	23.2 (1.8)	23.8 (1.7)	0.31	23.3 (1.8)	23.5 (1.7)	0.14	23.3 (1.8)	23.2 (1.7)	23.8 (1.7)	0.22
