# Supplementary material for: Analysis of Neuronal Excitability Profiles for Motor-Eloquent Brain Tumor Entities Using nTMS in 800 Patients
Source: Cancers (Basel). 2025 Mar 10;17(6):935. doi: 10.3390/cancers17060935 (PMC11940777; doi:10.3390/cancers17060935)
Supplement: Supplementary file 1 [file cancers-17-00935-s001.zip › TableS3.pdf]

Variable		Deficit (N = 267)	No Deficit (N = 530)	P
**Patient Charateristics**				
Female		122 (46%)	252 (48%)	0.621
Age (y)		58 (13)	50 (16)	<0.001
Antiepileptic Medication		116 (44%)	275 (52%)	0.021
**Tumor Location & Morphology**				
Motor Location	M1-TMS-Infiltration	127 (48%)	147 (28%)	<0.001
	TTD (mm)	5.1 (6.2)	7.6 (6.9)	<0.001
Dominant Hemisphere		116 (43%)	240 (45%)	0.622
Tumor Volume (ml)		23 (24)	22 (26)	0.757
Edema Volume (ml)		63 (48)	30 (40)	<0.001
Multifocal (≥2 Foci)		70 (26%)	108 (21%)	0.062
Tumor Recurrence		67 (25%)	118 (22%)	0.372
**Neuropathology**				
Tumor Entity	Glioma	133 (52%)	322 (62%)	
	Metastasis	101 (39%)	82 (16%)	
	Benign	23 (9%)	118 (23%)	<0.001
Glioma Type				
WHO Grade	WHO 2	10 (9%)	44 (15%)	
	WHO 3	13 (11%)	93 (31%)	
	WHO 4	92 (80%)	159 (54%)	<0.001
IDH Mutation		26 (23%)	164 (55%)	<0.001
1p19q Codeletion		11 (10%)	57 (19%)	0.018
Subtype	Oligodendroglioma	11 (10%)	57 (19%)	
	Astrocytoma	15 (13%)	107 (36%)	
	Glioblastoma	89 (77%)	132 (45%)	<0.001
**NTMS Parameter**				
RMT	Sick (V/m)	103 (33)	95 (23)	<0.001
	Healthy (V/m)	97 (24)	96 (22)	0.671
	Ratio (%)	110 (35)	101 (21)	<0.001
	Ratio (Pathologic)	153 (66%)	269 (57%)	0.017
Area	Sick (mm^2)	311 (224)	303 (221)	0.652
	Healthy (mm^2)	319 (258)	300 (228)	0.397
Amplitude	Sick (μV)	459 (475)	660 (630)	<0.001
	Healthy (μV)	710 (707)	800 (785)	0.207
Latency	Sick (ms)	23.9 (2.6)	23.2 (2.1)	<0.001
	Healthy (ms)	23.9 (2.0)	23.4 (1.9)	0.001
